# Differentiation of Th Subsets Inhibited by Nonstructural Proteins of Respiratory Syncytial Virus Is Mediated by Ubiquitination

**DOI:** 10.1371/journal.pone.0101469

**Published:** 2014-07-03

**Authors:** Ling Qin, Dan Peng, Chengping Hu, Yang Xiang, Yigang Zhou, Yurong Tan, Xiaoqun Qin

**Affiliations:** 1 Respiratory Department, Xiangya Hospital, Central South University, Changsha, Hunan, China; 2 Department of Basic Medicine, Xiangya School of Medicine, Central South University, Changsha, Hunan, China; University of Georgia, United States of America

## Abstract

Human respiratory syncytial virus (RSV), a major cause of severe respiratory diseases, constitutes an important risk factor for the development of subsequent asthma. However, the mechanism underlying RSV-induced asthma is poorly understood. Viral non-structural proteins NS1 and NS2 are critically required for RSV virulence; they strongly suppress IFN-mediated innate immunity of the host cells. In order to understand the effects of NS1 and NS2 on differentiation of Th subsets, we constructed lentiviral vectors of NS1 or NS2 to infect 16 HBE and analyzed the expression of HLA-DR, CD80 and CD86 and differentiation of Th1, Th2 and Th17 by Flow Cytometric Analysis and real-time PCR. The results showed that NS1 inhibited expression of HLA-DR, CD80 and CD86 and differentiation of Th1, Th2 and Th17 lymphocytes, which could be reversed by deleting elongin C binding domain. NS2 inhibited the differentiation of Th2 and Th17, which was reversed by proteasome inhibitors of PS-341. Our results indicated that NS1 inhibited the differentiation of T lymphocytes through its mono-ubiquitination to interacted proteins, while NS2 inhibited differentiation of Th2 and Th17 through ubiquitin-proteasome pathway, which may be related with the susceptibility to asthma after RSV infection.

## Introduction

Respiratory syncytial virus (RSV) is an important respiratory pathogen that produces an annual worldwide epidemic of respiratory illnesses primarily occurring in infants, but also in adults. However, the impact of RSV in the clinic reaches beyond infections, as it has been suggested that infection with RSV may cause a predisposition to the development of asthma [1]. Airway hyperactivity and related pathologic changes have been reported in RSV-infected animals in our previous studies [2]; however, the mechanisms underlying RSV-induced asthma are poorly understood.

The mechanisms underlying asthma which are considered important are classically characterized by immune activation and immune imbalance. In response to antigen stimulation, naive CD4^+^T cells differentiate into different subsets of Th cells that are classified based on distinct cytokine profiles and immune regulatory functions [3]–[4]. Th1 cells produce IL-2 and IFN-γ, and play an important role in cell-mediated immune responses against intracellular pathogens. Th2 cells produce IL-4, IL-5 and IL-10 and Th17 cells produce IL-17. Th2 and Th17 subsets are both involved in humoral immunity and allergic responses, such as asthma [5].

RSV has a single-strand negative-sense RNA genome and encodes 11 proteins. The two nonstructural proteins (NS) of human RSV have recently drawn attention because of their potential accessory function. As early synthesized viral proteins, NS1 and NS2 of RSV provide not only the necessarily enzymatic activities in viral replication, but also enzymatic activities to their interacted proteins in host cells, inducing immune compromise of epithelial functions and further development of bronchiolitis and asthma [6]. Various lines of experimental evidence have clearly established a role for the NS proteins in multiple members of the cellular interferon pathways [7].

Airway epithelial cells are closely related to asthma, and damage of airway epithelial structure and function may result in susceptibility to asthma, which could be a priming process in asthma [8]. Epithelial cells also play an important role in immunologic imbalance of the respiratory system, particularly in the case of RSV infection, since this virus productively replicates only in the respiratory mucosa [9]–[10]. Therefore, we hypothesize that viral non-structural proteins NS1 and NS2 may induce abnormal signal transduction of bronchial epithelial cells through their enzymatic activities, thereby driving the abnormal differentiation of Th subsets of CD4^+^ T lymphocytes. The aim of this study was to determine the differentiation of CD4^+^ T lymphocytes and its mechanisms *in vitro* when human bronchial epithelial cells (16- HBE) were infected by lentiviral vectors of NS1 or NS2.

## Methods

### Production of lentiviral vectors

The full coding sequence of NS1 with an insert of flag after ATG was cloned into EcoR I and BamH I of plasmid pLenO-GFP to construct pLenO-GFP-NS1. PLenO-GFP-mNS1 was constructed as above by deleting the elongin C binding domain of NS1. The full coding sequence of NS2 with an insert of HA after ATG were cloned into EcoR I and Not I of plasmid pLenO-RFP to construct pLenO-RFP-NS2 (Genscript, Nanjing, China). The constructed plasmids were verified by restriction enzyme mapping and direct DNA sequencing. Production of lentivirus was performed using the combined ratio of transfer plasmid, packaging plasmid, Env plasmid and pRSV-Rev plasmid at 4:2:1:1 by using CaCl_2_ (Sigma). A collection was made after 48 hrs and was cleared by centrifugation at 1500 rpm for 5 min at 4°C then passed through a 0.45 µm pore PVDF Millex-HV filter (Millipore). Concentration of lentivirus using ultracentrifugation was performed for 90 min at 90,000 g. Supernatant was completely removed and virus pellets resuspended in 300 µL PBS overnight at 4°C and stored at −80°C until use.

Titers were determined by transducing 1×10^5^ of HEK293T cells with different concentrations of lentiviral vectors in 100 µL of RPMI 1640 growth medium. The following day, supernatants were removed and fresh growth media were added. After a further incubation for 4 days, cells were harvested and determined by fluorescence microscopy and flow cytometry (BD Pharmingen). Titers were determined based on the percentage of positive cells and calculated according to the following formula (F×N×D×1,000)/V, where F  =  percentage of positive cells, N  =  number of cells at time of transduction (usually 1×10^5^ cells), D  =  fold dilution of vector used in transduction, V  =  volume (µL) of diluted vector sample in each well. The virus titer is expressed as transducing units/mL (TU/mL) ± standard error.

### Cell culture and lentiviral transduction of 16HBE

16HBE cells were cultured in DMEM supplemented with 10% FBS at 37°C under 5% CO_2_ in humidified air. 60–70% confluent monolayer cultures of 16 HBE were infected with lentivirus at MOI of 10 according to previous experiments. After incubation overnight, lentiviral vectors were removed and fresh media were added. Following 48 hrs of culture, the cells were removed and positive cells were determined by fluorescence microscopy or indirect immnofluoresent technology.

### Assay of NS1 and NS2 by indirect immnofluoresence

16HBE were fixed by 95% ethanol and 0.1% Triton-X100. The fixed slides were incubated with 3% H_2_O_2_, and blocked with a normal goat serum for 20 min. The slides were then incubated with a mouse anti-human flag or HA monoclonal antibody (1:200) at 4°C for overnight. The Cy3-labeled or FITC-labeled secondary antibody(1:250) was added to the slides respectively at 37°C for 2 h. The slides were finally mounted with mounting fluid and examined by fluorescence microscopy.

### Assay of HLA-DR, CD80 and CD86 by Flow Cytometric Analysis

The monoclonal antibodies used for flow cytometry were monoclonal anti-human HLA-DR, CD80 and CD86 (Biolegend). After being treated with OVA (1 mg/mL) for 1 hour or OVA (1 mg/mL) plus PS-341 (50 nM) for 1 hour, 10^6^ of 16 HBE were incubated with 10 uL of monoclonal antibody at room temperature for 20 min. The cells were then washed twice and resuspended in PBS containing 1% FBS and 0.1% NaN3 (Sigma) and immediately analyzed with FACS (Becton Dickinson, USA). Isotype-matched antibodies were used as controls. The levels of antigen expression were expressed as a percentage of positive cells in the total cells.

### Co-culture of 16HBE and Jurkat E6-1

Jurkat E6-1 (CD4^+^ T cells, a gift from Yao Xiaojian, Microbiology Department of Manitoba University, Manitoba, Canada) were cultured in 1640 medium supplemented with 10% FBS. In co-cultured experiments, 16HBE were located at the bottom of the culture plate and the lymphocytes were suspended in culture medium. Mock-infected and lentivirus-infected 16HBE were plated in 6-well culture plates with 2×10^6^ cells/well. After 16 HBE adhered to the bottom of the culture plate for 12 h, lymphocytes were added to the 16 HBE in a 1:1 ratio. 24 hrs later 8 ul of PMA/lonomycin and 8 ul of BFA/Monensin mixture were added to the co-cultured system. After continuous incubation for 24 hrs, the CD4^+^ T cells and supernatants were collected respectively.

### Assay of Th subsets by flow cytometry analysis

PE anti-human IL-4, PE anti-human IL-17, PE anti-human IFN-γ were obtained from eBiosicence (USA) and used as staining reagents. For intracellular staining of IL-4, IFN-γ and IL-17, lymphocytes were pre-conditioned in the presence of 2 µL of monensin (×1000; Biolengend, USA) for 6 h, which can inhibit secretion of newly produced cytokines. Then, lymphocytes were incubated with monoclonal antibody at room temperature for 20 min. The cells were then washed twice and resuspended in PBS containing 1% FBS and 0.1% NaN3 (Sigma) and immediately analyzed with FACS (Becton Dickinson, USA). Isotype-matched antibodies were used as controls. The levels of antigen expression were expressed as a percentage of positive cells in the total cell**s.**

### Assay of Th subsets by real-time PCR

RNA was isolated and reverse transcribed into cDNA and analyzed by quantitative real-time PCR with SYBR Green I. The primers for T-bet were 5′- GAGTTTCGAGCAGTCAGCA-3 and 5′- CGGTGTCCTCCAACCTAAT-3′, 471 bp; for GATA-3 were 5′ -CCCGCACCTCTTCACCTT-3′and 5′- CCTGGTACTTGAGGCACTC-3′, 122 bp; for RoR
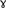
t were 5′ -TTTCCGAGGATGAGATTGC-3′and 5′-AGCGGCTTGGACCACGAT-3′, 257 bp. Briefly, 2 µl (out of 20 µl) of the reverse-transcribed reaction mix was added to a 50 µl PCR mixture for 35 cycles. Each cycle included 94°C for 20 seconds,53°C for 30 seconds and 72°Cfor 30 seconds. Raw data were normalized to β- actin (5′- TGACGTGGACATCCGCAAAG-3′and 5′- CTGGAAGGTGGACAGCGAGG-3′).

### Cytokine measurement

Human IFN-γ, IL-4 and IL-17 concentrations in co-cultured supernatants were determined using ELISA kits from R&D Systems (USA).

### Statistical analysis

Data are presented as the mean±SEM. Statistical analysis of 2-way ANOVA was used to analyse the release of cytokines from lymphocytes in the co-cultured system. Univariate ANOVA, followed by Dunnett's t-test, was used to analyze Th subset distribution. Two-tailed and paired tests were used throughout the whole statistic. A *P* value<0.05 was considered statistically significant. Statistics were calculated by SPSS/Windows version 13.0 software.

## Results

### Lentiviral construction and infection of 16HBE

**Figure 1:**
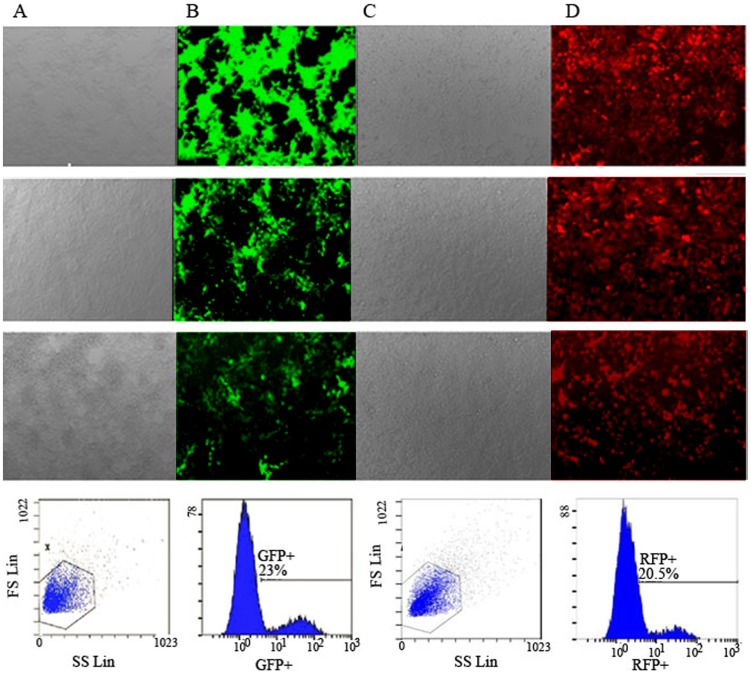
Titers of pLenO-GFP-NS1 and pLenO-RFP-NS2 were assayed by flow cytometry. A, B: Infection of HEK293T cells with different dilutions of pLenO-GFP-NS1 under light microcopy and fluorescence microscopy respectively (×100); C, D: Infection of HEK293T cells with different dilutions of pLenO-RFP-NS2 under light microcopy and fluorescence microscopy respectively. From above to below represent the concentration of 1.0 ul, 0.1 ul, 0.01 ul and representative images of flow cytometry (0.01 ul).

**Figure 2:**
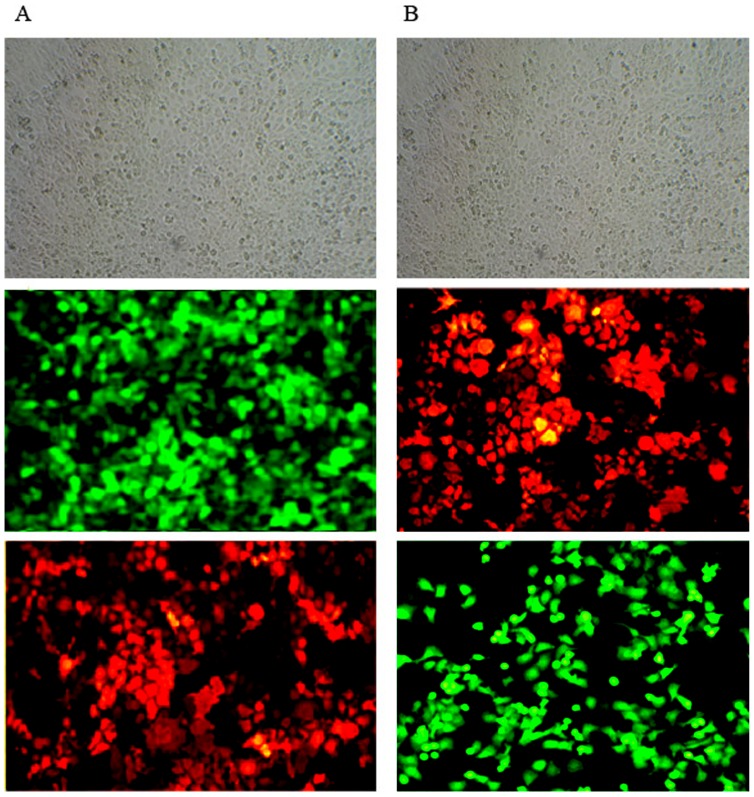
Efficiencies of lentiviral infections to 16 HBE were assayed by fluorescence microscopy and indirect immunofluorescent technology. A: Infection of 16 HBE by pLenO-GFP-NS1 (MOI = 10); B: Infection of 16 HBE by pLenO-RFP-NS2 (MOI = 10). From above to below represent images under light microcopy, fluorescence microscopy and indirect immunofluorescent assay (×200).

The pLenO-GFP-NS1, pLenO-GFP-mNS1 and pLenO-RFP-NS2 were verified by restriction enzyme mapping and direct DNA sequencing respectively (data not shown). The titers were determined by transducing HEK293T cells with different dilutions of lentiviral vectors and subsequent flow cytometry. The results showed that the titer of GFP-NS1^+^ is 2.3×10^9^ TU/ml and RFP-NS2^+^ is 2.1×10^9^ TU/ml (figure 1). Then, the expression of NS1 or NS2 was assayed by fluorescence microscopy and indirect immnofluoresent technology. Under fluorescence microscopy, different MOIs were tested. When MOI was equal to 10, the measured efficiency of lentiviral infection was above 80% (Figure 2). So this concentration was chosen in next assays. By using indirect immnofluoresent technology, NS1 was conjugated with Cy3, which was indicated by red fluorescence distributed within the cells, while NS2 was conjugated with FITC, bright yellow green fluorescence within the cells (figure 2).

### NS1 inhibited the expression of CD80, CD86 and HLA-DR through its elongin C binding domain

**Figure 3:**
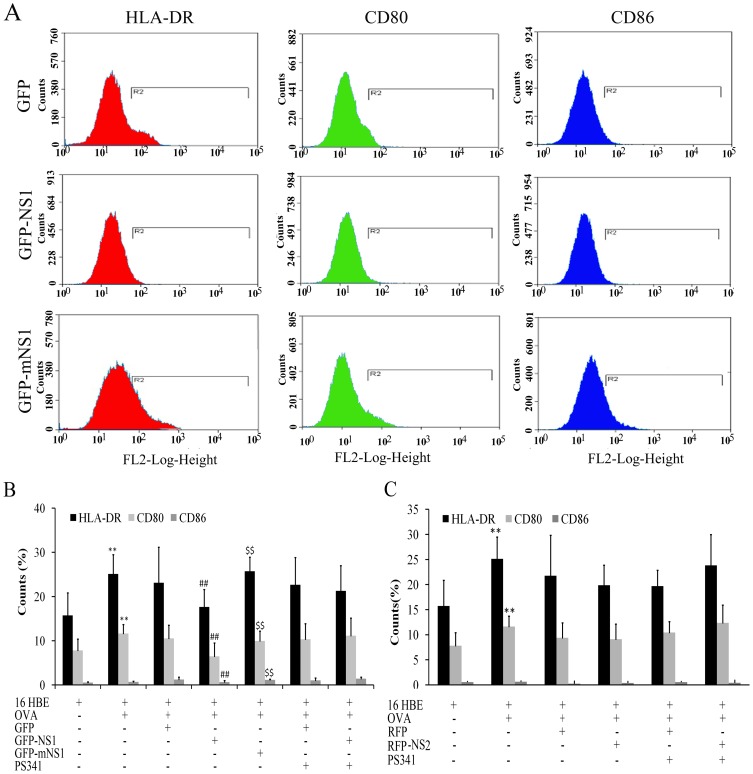
The expression of HLA-DR, CD80 and CD86 on 16 HBE was assayed by flow cytometry. A, B: The influence of NS1 on the expression of HLA-DR, CD80 and CD86 on 16 HBE with or without OVA treatment (**P<0.01 versus 16 HBE group; ##P<0.01 versus GFP group; $$P<0.01 versus GFP-NS1 group). C: The influence of NS2 on the expression of HLA-DR, CD80 and CD86 on 16 HBE with or without OVA (**P<0.01 versus 16 HBE group). Data represent Means ± SE of 6 experiments.

Constitutive expression of HLA-DR, CD80 (B7-1) and CD86 (B7-2) molecules was observed on 16HBE. HLA-DR and CD80 molecules were upregulated in the presence of OVA, while CD86 was not upregulated (Fig3 A, B). NS1 inhibited the expression of HLA-DR (P = 0.003), CD80 (P = 0.004) and CD86 (P = 0.010) molecules in the presence of OVA (Fig3 A, B). The expression of HLA-DR, CD80 and CD86 was reversed by treatment 16HBE with pLenO-GFP-mNS1, but not 50 nM of PS-341 (a potent and specific inhibitor of the ubiquitin-proteasome). NS2 had no influence on the expression of CD80, CD86 and HLA-DR molecules in the presence of OVA (Fig3C).

### NS1 inhibited differentiation of Th1, Th2 and Th17 through its elongin C binding domain

**Figure 4:**
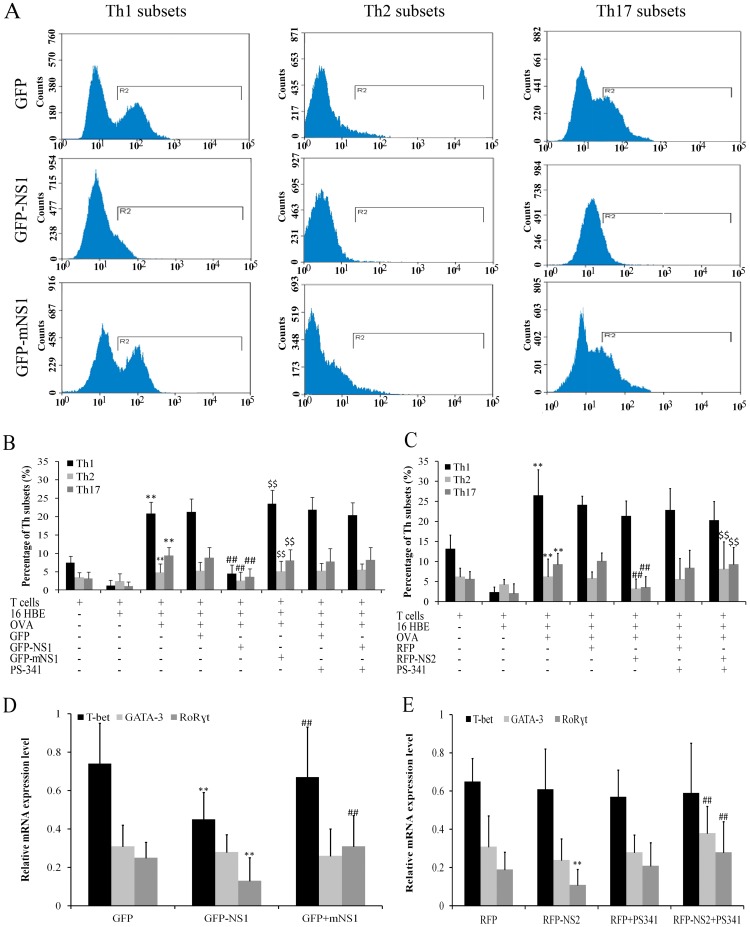
The changes of Th subsets were assayed by flow cytometry and real-time PCR. A, B: Influence of NS1 on changes of Th subsets was assayed by flow cytometry (**P<0.01 versus 16 HBE group; ##P<0.01 versus GFP group; $$P<0.01 GFP-NS1 group). C. Influence of NS2 on changes of Th subsets was assayed by flow cytometry (**P<0.01 versus 16 HBE group; ##P<0.01 versus RFP group; $$P<0.01 RFP-NS2 group). D: Influence of NS1 on changes of Th subsets was assayed by real-time PCR (**P<0.01 versus GFP group; ##P<0.01 GFP-NS1 group). E. influence of NS2 on changes of Th subsets was assayed by real-time PCR (**P<0.01 versus RFP group; ##P<0.01 RFP-NS2 group). Data represent Means ± SE of 6 experiments.

The distribution of Th subsets was analyzed after 16 HBE were infected with pLenO-GFP-NS1, pLenO-GFP-mNS1 or pLenO-GFP control vector and co-cultured with CD4^+^ T cells for 48 h. The results showed that differentiation of Th1 (*P* = 0.001), Th2 (*P* = 0.007) and Th17 (*P* = 0.001) was inhibited by treatment 16 HBE with pLenO-GFP-NS1 when compared with the empty vector group. The effects disappeared by treatment 16 HBE with pLenO-GFP-mNS1. However, differentiation of Th1, Th2 and Th17 can not be reversed by treatment pLenO-GFP-NS1 –infected 16 HBE with 50 nM of PS-341 (Figures 4 A, B).

**Figure 5:**
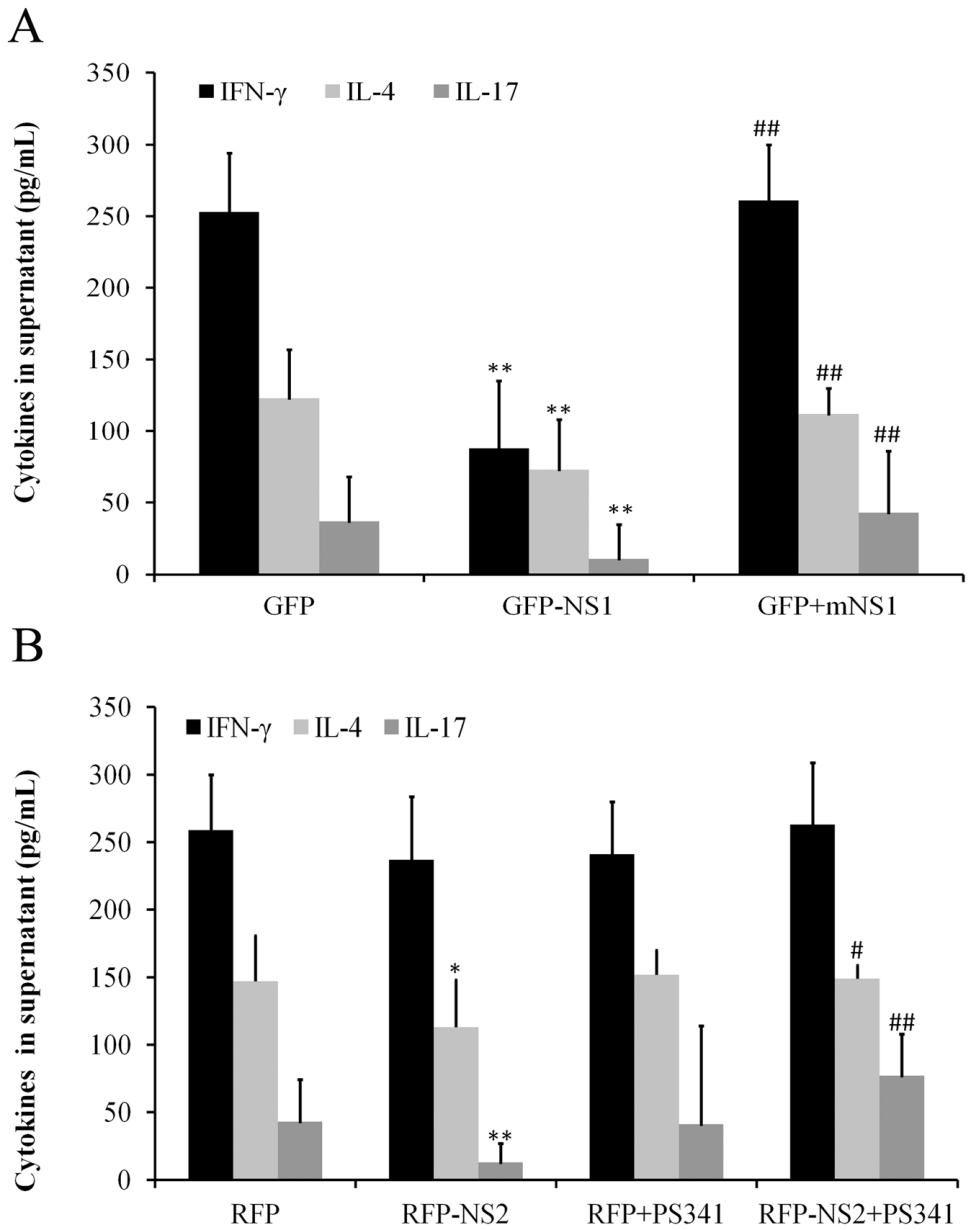
The contents of IFN-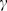, IL-4 and IL-17 from cocultured 16 HBE and lymphocytes were assayed by ELISA (n = 6). A: Influence of NS1 on the secretion of IFN-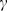, IL-4 and IL-17 from cocultured 16 HBE in the presence of OVA and lymphocytes (**P<0.01 versus GFP group; ## P<0.01 versus GFP-NS1 group) B: Influence of NS1 on the secretion of IFN-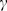, IL-4 and IL-17 from cocultured 16 HBE in the presence of OVA and lymphocytes. (*P<0.05, **P<0.01 versus RFP group; # p<0.05, ## P<0.01 versus RFP-NS2 group).

By using real-time PCR, we observed that NS1 decreased the expression of T-bet and RoR
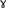
t. The effects can be cancelled by mNS-1 but not PS-341 (Figure 4 D). Lymphocytes in the resting state released low levels of cytokines. IFN-γ was upregulated in the presence of OVA (data not shown). After co-culture with pLenO-GFP-NS1 infected 16 HBE (with OVA) for 48 h, the release of cytokines IFN-γ, IL-4 and IL-17 from lymphocytes significantly decreased when compared with those in control empty vector group. The effects of NS1 can be cancelled by treatment 16 HBE with pLenO-GFP-mNS1, but not 50 nM of PS-341 (Figure 5A).

### NS2 inhibited Th2 and Th17 differentiation through ubiquitin-proteasome pathway

The distribution of Th subsets was analyzed after 16 HBE were infected with pLenO-RFP-NS2 or its corresponding control vector and then co-cultured with CD4^+^ T cells for 48 h. The results showed that differentiation of Th2 (*P* = 0.010) and Th17 (*P* = 0.008) was inhibited by treatment with pLenO-RFP-NS2 pre-interfered 16 HBE when compared with empty vector group. However, differentiation of Th2 and Th17 can be reversed by treatment with pLenO-RFP-NS2 and 50 nM of PS-341 pre-interfered 16 HBE (Figures 4C).

Results from real-time PCR also showed that NS2 decreased the expression of RoR
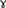
t. The expression of GATA-3 and RoR
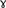
t in CD4^+^ T cells can be induced by treatment with pLenO-RFP-NS2 and 50 nM of PS-341 pre-interfered 16 HBE (Figures 4E). After co-culture with pLenO-RFP-NS2-infected 16 HBE for 48 h, the release of cytokines IL-4 (*P* = 0.046) and IL-17 (*P* = 0.008) from lymphocytes significantly decreased when compared with empty vector group. The effects of NS2 can be reversed by 50 nM of PS-341 (Figure 5B).

## Discussion

Airway epithelial cells have the potential for presenting antigens, releasing inflammatory mediators, and activating the immune response, and at the same time are main targets of RSV infection. Thus, we speculate that RSV infections induce airway inflammation or aberrant immune reactions through changing functions of airway epithelial cells, which may be related with enzymatic activities of non-structural proteins.

When subjected to the invasion of external antigens (bacteria or viruses), the human body will initiate immune protection against pathogens. Exogenous microbial antigens are taken up by antigen presenting cells (APCs) and presented to lymphocytes, which triggers immune responses. Proliferation and activation of lymphocytes are in response to Th cell differentiation and the release of large amounts of cytokines. Cytokines also promote source lymphocytes and other immune cells to further differentiate and proliferate, and subsequently generate a wide range of immune effector cells [11]. The antigen signals which triggered activation of lymphocytes might be processed and synthesized by bronchial epithelial cells which absorbed antigens, thereby activating the function of lymphocytes in immune responses. The activation and differentiation of T cells require a specific antigen peptide-MHCII molecule complex (first signal) which is processed and synthesized by APCs. In addition to the first signal, CD80 and CD86 and other co-stimulatory molecules combined with CD28 expressed by T cells are the second signal to activate T cells. It has been shown that bronchial epithelial cells in stress or pathologic conditions can express MHCI and MHCII molecules, and then mediate T cell migration [12]. In animal models of asthma, HLA-DR and HSP70, two types of molecules with antigenic characteristics, are over-expressed in airway epithelium [13]. Papi et al [14] reported that B7-1 and B7-2 co-stimulatory molecules are expressed in lung cancer cell lines and bronchial epithelial cells after infection with rhinovirus. Therefore, the bronchial epithelial cells are considered to have potential antigen-presenting functions that have early contact with pathogenic antigen and initiate specific immune responses. In this study, 16 HBE absorbed OVA proteins first, then processed and synthesized antigen-presenting complex as the first signal, as well as expressed co-stimulatory molecules as the second signal, which comprised the activation signals of lymphocytes. NS1 or NS2 has different effects on these processes. NS1 inhibited the expression of CD80, CD86 and HLA, while NS2 had no influence on the expression of CD80, CD86 and HLA, indicating that NS1 has the potential to inhibit the activation and differentiation of T cells.

RSV infection may increase the risk of asthma morbidity, but the underlying mechanism remains unknown. In allergic asthma, a critical event is the activation of Th cells, leading to a predominance of Th2 cytokines over Th1 cytokines [15]. T cell subsets, in addition to Th1 and Th2, Th17 cells also have been identified in asthma [16]. Among many influential factors, the cytokine microenvironment, in which Th0 cells contact APCs, is important in regulating Th cell differentiation. For example, IL-12 and IFN-γ promote Th0 cells to differentiate to Th1 cells, and IL-4 promotes Th0 cells to differentiate to Th2 cells. Jurkat E6-1 is from Human T cell leukemia. The cells are CD4+ and can release interferon-γ [17]. According to our experiments, although they have low ability of differentiation, they can be induced to secrete interferon-γ, IL-4 and IL-17. Our results showed that 16 HBE treated with OVA induced Jurkat E6-1 to secrete large quantities of IFN-γ, which induced differentiation of Th1-dominated subset. NS1 inhibited the proliferation of Th1, Th2 and Th17 through its elongin C binding domain while NS2 inhibited differentiation of Th2 and Th17 through ubiquitin-proteasome pathway. Moreover, we provide further evidence of abnormal differentiation of Th cells by assays of IFN-γ, IL-4 and IL-17 in secreted supernatants from co-cultured system.

Th2 and Th17 subsets are both involved in humoral immunity and allergic responses. Previous study by Munir S reported that RSV NS1 protein suppressed proliferation of Th17 cells, and promoted proliferation of Th2 cells [18].However, in present study, no evidences showed that NS1 promoted proliferation of Th2. The differences may be caused by the different T cell strain. We will further probe the mechanism underlying the differences.

In previous study [19], our results showed that 16 HBE with prolonged RSV infection induced excessive Th2 and Th17 responses which could be the result of an altered pattern of Th subset differentiation after stimulation by RSV-infected epithelial cells. In present study, we observed that NS1 and NS2 protein inhibited Th2 and Th17 responses, indicating that RSV and its nonstructural proteins NS1 and NS2 have different effects on differentiation of CD4^+^ T cells. NS1 and NS2 induced the persistent infection by inhibiting the immunity which maybe related with asthma pathogenesis.

In previous study [20], we observed that NS1 induced monoubiquitination of H2BD, and this effect was related with the activity of elongin C binding domain of NS1. In present study, we also observed that the effects of NS1 were related with its activity of using the elongin-culin E3 ligase, indicating that NS1 has the activity of mono-ubiquitination to the interacted proteins. The proteasome is a self-compartmentalizing, multimeric protease that belongs to the ubiquitin-proteasome system (UPS) for intracellular protein degradation. The majority of short-lived but also long-lived proteins are substrates for the proteasome, highlighting the importance of this degradation machinery in many cellular regulatory mechanisms. The effects of NS2 can be reversed by proteasome inhibitor PS341, indicating that NS2 inhibited differentiation of Th2 and Th17 through ubiquitin-proteasome pathway.

Our data suggest that susceptibility to asthma after RSV infection would be related to lymphocyte inhibition and immune compromise caused by NS1 and NS2. Bronchial epithelial cells play a key role by presenting inhibiting signals, so inhibiting the differentiation of CD4^+^T cells after RSV infection.
